# Human Cytochrome P450 3A4 as a Biocatalyst: Effects of the Engineered Linker in Modulation of Coupling Efficiency in 3A4-BMR Chimeras

**DOI:** 10.3389/fphar.2017.00121

**Published:** 2017-03-21

**Authors:** Danilo Degregorio, Serena D'Avino, Silvia Castrignanò, Giovanna Di Nardo, Sheila J. Sadeghi, Gianluca Catucci, Gianfranco Gilardi

**Affiliations:** Department of Life Sciences and Systems Biology, University of TurinTurin, Italy

**Keywords:** P450 biocatalysis, domain interaction, glycine linker, stability, uncoupling, electron transfer

## Abstract

Human liver cytochrome P450 3A4 is the main enzyme involved in drug metabolism. This makes it an attractive target for biocatalytic applications, such as the synthesis of pharmaceuticals and drug metabolites. However, its poor solubility, stability and low coupling have limited its application in the biotechnological context. We previously demonstrated that the solubility of P450 3A4 can be increased by creating fusion proteins between the reductase from *Bacillus megaterium* BM3 (BMR) and the N-terminally modified P450 3A4 (3A4-BMR). In this work, we aim at increasing stability and coupling efficiency by varying the length of the loop connecting the two domains to allow higher inter-domain flexibility, optimizing the interaction between the domains. Starting from the construct 3A4-BMR containing the short linker Pro-Ser-Arg, two constructs were generated by introducing a 3 and 5 glycine hinge (3A4-3GLY-BMR and 3A4-5GLY-BMR). The three fusion proteins show the typical absorbance at 450 nm of the reduced heme-CO adduct as well as the correct incorporation of the FAD and FMN cofactors. Each of the three chimeric proteins were more stable than P450 3A4 alone. Moreover, the 3A4-BMR-3-GLY enzyme showed the highest NADPH oxidation rate in line with the most positive reduction potential. On the other hand, the 3A4-BMR-5-GLY fusion protein showed a V_max_ increased by 2-fold as well as a higher coupling efficiency when compared to 3A4-BMR in the hydroxylation of the marker substrate testosterone. This protein also showed the highest rate value of cytochrome *c* reduction when this external electron acceptor is used to intercept electrons from BMR to P450. The data suggest that the flexibility and the interaction between domains in the chimeric proteins is a key parameter to improve turnover and coupling efficiency. These findings provide important guidelines in engineering catalytically self-sufficient human P450 for applications in biocatalysis.

## Introduction

Cytochromes P450 are heme-thiolate monooxygenases found in all domains of life (Coon, 2005; Bernhardt, 2006) able to metabolize several endogenous molecules and xenobiotics, including drugs (Nelson et al., 1996; Ortiz de Montellano, 2005; Isin and Guengerich, 2007).

They catalyze many chemical reactions such as hydroxylation of non-activated carbon atoms, epoxidation of double bonds and dealkylation on C-, N-, and S- atoms (Isin and Guengerich, 2007). Electrons for these reactions are provided by NADPH and transferred to the heme cofactor via different redox partners classified by Hannemann et al. (2007). Thus, NADPH is used as electron source and in the presence of molecular oxygen, reaction product(s) can be generated in single or multistep reactions.

Although the human liver enzymes are an attractive biotechnological target in their use as biocatalysts for their ability to produce pharmaceuticals and drug metabolites, limitations are given by poor stability and by the so-called “uncoupling,” the formation of reactive oxygen species at the heme site leading to the release of intermediates such as superoxide and peroxide during the reaction cycle (Guengerich, 2002; Meunier et al., 2004; Zangar et al., 2004; Denisov et al., 2005). The uncoupling process can lead to a loss of NADPH redox equivalents as high as 99% in human cytochrome P450 1A2 (Mayuzumi et al., 1993) or 16% for 3A4 (Perret and Pompon, 1998) and it can be seen as a wasteful process. These high uncoupling levels, together with the need of a reductase, are a considerable disadvantage in the use of human enzymes as biocatalysts.

On the other hand, bacterial P450 enzymes are known to be well coupled and as in the case of P450 BM3 from *Bacillus megaterium*, have been shown to be able to produce drug metabolites typical of the human enzymes with a high coupling efficiency (Di Nardo et al., 2007; Di Nardo and Gilardi, 2012). This has led to a wide range of protein engineering studies on this enzyme to widen its catalytic abilities (Tsotsou et al., 2012, 2013; Ryu et al., 2014; Di Nardo et al., 2015; Ren et al., 2015; Capoferri et al., 2016).

One factor that makes P450 BM3 a well-coupled biocatalyst resides in the productive interaction between the heme catalytic domain (BMP) and its flavin-containing reductase domain (BMR) that, unlike in other cytochromes P450, are all part of the same polypetide chain. For this reason we already reported the construction of different fusion human P450 enzymes, engineered by connecting the reductase domain BMR with human cytochromes P450 2E1(Fairhead et al., 2005), 3A4, 2C9, 2C19 (Dodhia et al., 2006), 2A6, CYP2C6, and CYP4F11 (Ortolani, 2012; Rua, 2012a; Castrignanò et al., 2014), monkey 2C20 (Rua et al., 2012b) and dog CYP2D15 (Sadeghi and Gilardi, 2013; Rua et al., 2015).

As human cytochrome P450 3A4 is responsible for the metabolism of the majority of commercial drugs and it is at the origin of many drug-drug interactions of clinical concern (Evans and Relling, 1999), we further characterized the parameters influencing uncoupling in the reconstituted fusion protein P450 3A4-apo-BMR, such as flavin content, substrate, and NADPH concentration, pH and ionic strength (Degregorio et al., 2011). Among P450 systems, P450 BM3 is considered as a model for self-sufficiency and high coupling efficiency (Munro et al., 1996; Noble et al., 1999). In 2005, Neeli and colleagues suggested that BM3 fatty acid hydroxylation activity can be related to a dimeric form of the enzyme and for this protein electron transfer is achieved through an “inter subunits” interaction between protomers rather than direct flow between the di-flavin and the heme held by the same polypeptide chain (Neeli et al., 2005).

In the present work we consider another parameter that can influence the coupling efficiency in the fusion protein P450 3A4-BMR: the length of the loop linking the oxygenase domain with the reductase one. In 1995, Govindaraj and Poulos demonstrated that the length of the linker is the critical feature that controls flavin-to-heme electron transfer in cytochrome P450 BM3 (Govindaraj and Poulos, 1995). Moreover, the role of the linker sequence in facilitating the ability of cyt *b*_5_ to reduce P450 2B4 was explored by altering the length and sequence of the flexible linker region between the heme domain and membrane anchor of cyt *b*_5_ (Clarke et al., 2004). Clarke and co-workers demonstrated that, although the sequence of the linker region can be varied without significant effects on the properties of cyt *b*_5_, the linker region has a critical minimum length of 6–8 residues.

Here we aim at elucidating first if then how the length of the linker connecting the reductase and catalytic domain in the 3A4-BMR fusion protein has an effect in modulating the activity and the uncoupling process.

**Figure 1:**
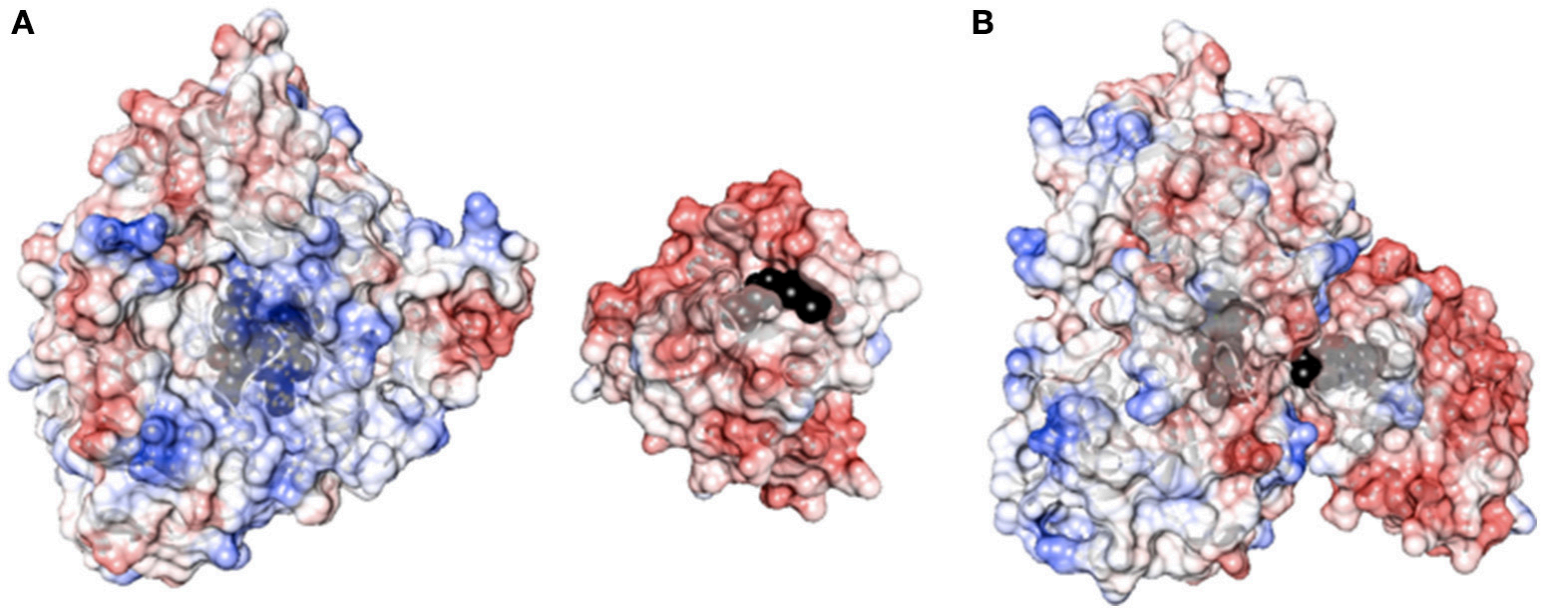
**(A)** Surface charge distribution of P450 3A4 (left, PDB ID 1TQN) and the FMN binding domain of P450 BM3 (right, PDB ID 1BVY). Heme and FMN are shown in black. **(B)** Complex between P450 3A4 and the FMN binding domain of BMR. The complex was created by super-imposition of the crystal structure of P450 3A4 with the heme domain of P450 BM3 in the complex crystal structure.

Flexibility between protein domains is a critical parameter in modulating the uncoupling process via the formation of an active complex and by tuning the electron flow to the heme. The ability of the reductase of P450 BM3 from *B. megaterium* (BMR) to transfer electrons in the redox chain from NADPH to the heme is evaluated by varying the length of the linker region between BMR and the N-terminally modified 3A4 heme domain. As shown in Figure 1, the BMR and P450 3A4 domains possess the surface charges complementarity that is known to be more crucial for the complex formation than when the physiological redox partner, cytochrome P450 reductase (hCPR) was used instead. In order for catalysis to occur, this active complex should form with high propensity. Thus, starting from the 3A4-BMR chimera in which the BMR domain is linked to the 3A4 *via* the short linker Pro-Ser-Arg, two new constructs are engineered to include 3- or 5-Gly inserted after the Pro-Ser-Arg loop leading to 3A4-3GLY-BMR and 3A4-5GLY-BMR chimeric proteins. This glycine rich region close to the C-terminal of P450 3A4 has been proposed to function as a flexible hinge that can provide the enzyme the degrees of freedom required to achieve a functional complex. The effect of the linker length on the electron transfer between the reductase and the catalytic domains in the presence of NADPH as electron supplier is studied for the three chimeras.

The electrons delivery from NADPH to the flavins of the reductase domainis is evaluated in the three complexes by anaerobic stopped flow spectroscopy measurement of NADPH consumption rate in the absence of substrate. The electron transfer capability of reducing equivalents by flavins of the reductase domain is investigated using cyt *c* as an artificial electron acceptor (Guengerich et al., 2009) and the effect of the linker length is examined. Finally, the ability of the BMR to sustain catalysis at the 3A4 catalytic domain is explored in relation to the length of the linker by investigating enzyme activity and coupling efficiency during erythromycin N-demethylation and testosterone 6β-hydroxylation.

## Materials and methods

### Chemicals and reagents

The pCW-3A4-BMR plasmid containing the gene of human P450 3A4 fused to the reductase of cytochrome P450 BM3 from *B. megaterium* (BMR) linked via a Pro-Ser-Arg linker was available in our laboratory (Dodhia et al., 2006). GenElute plasmid miniprep kit was obtained from Sigma (Italy). PCR purification kit and gel extraction kit were purchased from Macherey-Nagel (Germany).

The primers for PCR and oligonucleotides for the linker region between P450 3A4 and BMR reductase were prepared by MWG (Germany). DNA Ladder, restriction endonucleases enzymes *Avr II* and *Hind III*, Vent polymerase, T4 DNA ligase enzyme and buffers used in sub-cloning were from New England Biolabs (UK) and Promega (Italy). Horse heart cytochrome *c*, erythromycin, testosterone, 6β-hydroxytestosterone, horseradish peroxidase type X, superoxide dismutase from bovine erythrocytes, catalase, N,N-dimethylaniline, and 4-amino-2,3-dimethyl-1-phenyl-3-pyrazolin-5-one were purchased from Sigma (Italy). NADPH (tetrasodium salt) was acquired from Calbiochem (Germany). Human CPR was purchased from Life Technologies. All other chemicals, biochemicals used in this study were purchased from Sigma (Italy) at the highest available grade and used as recommended by the manufacturer.

### Engineering the linkers in the 3A4-BMR chimeras

**Figure 2:**
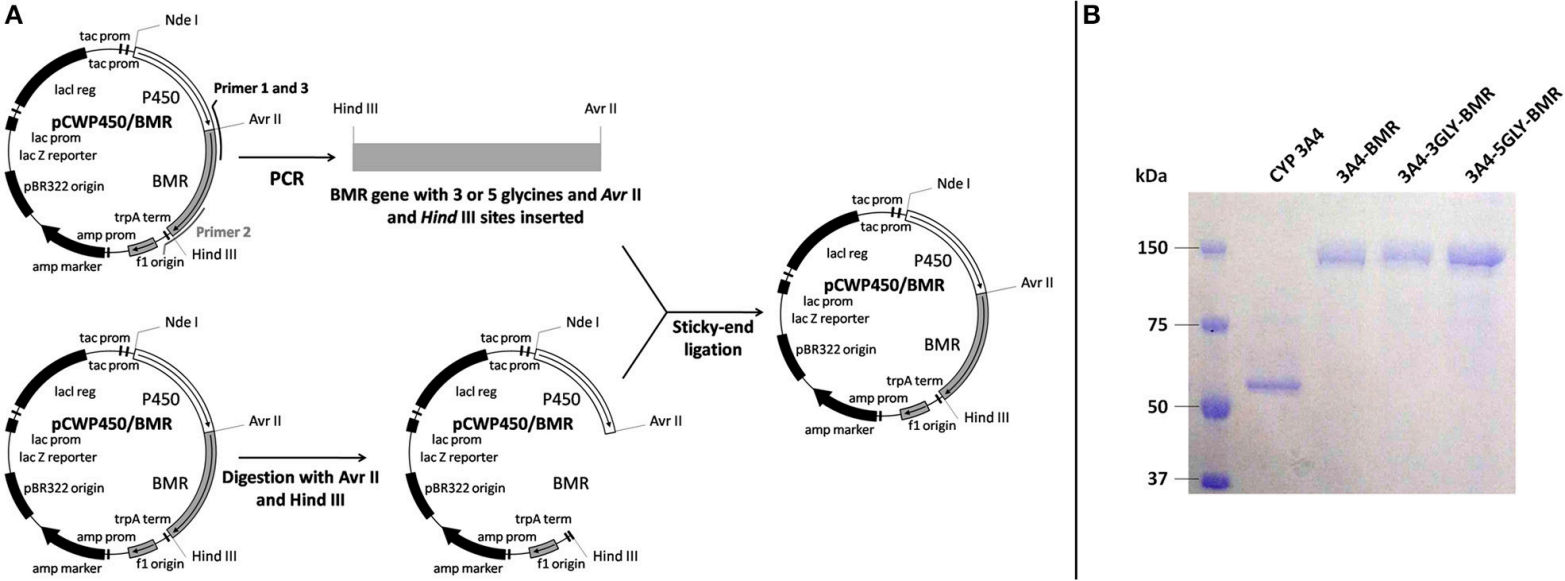
**(A)** Schematic illustration of the chimeras construction strategy. **(B)** SDS-PAGE of the purified chimeric proteins.

The strategy used in the construction of the fusion proteins is based upon introduction of 3 or 5 glycines in the pre-existent Pro-Ser-Arg linker connecting P450 CYP 3A4 domain and BMR one domain via 3 amino acids (Pro-Ser-Arg) by using as a starting template the vector pCW-3A4-BMR available in our laboratory (Figure 2A; Dodhia et al., 2006). Glycine was chosen to allow the highest flexibility of the loop to support conformational rearrangements between the two domains as much as possible.

All PCR reactions were performed in a DNA thermal cycler (PerkinElmer Life Sciences, Italy) and employed Vent polymerase under conditions recommended by the manufacturer (Promega, Italy).

The 3A4-3GLY-BMR chimera was engineered to contain the N-terminally modified human P450 CYP 3A4 (aa 13-503), the linker Pro-Ser-Arg-Gly-Gly-Gly and the C-terminal reductase (aa 472-1049) of P450 BM3 from *B. megaterium*. The 3A4-5GLY-BMR chimera contains the N-terminally modified human P450 3A4 (aa 13-503), the linker Pro-Ser-Arg-Gly-Gly-Gly-Gly-Gly and the C-terminal reductase domain (aa 472-1049) of *B. megaterium*. The gene coding for the BMR reductase was amplified using the forward 5-AGTGGAGCCCCTAGGTCAGGCGGTGGCAAAAAGGCAGAAAACGCTCATAAT-3′ and reverse 5′-GCTCATGTTTGACAGCTTATCATCGATAAGCTTTTACCC-3′ primers for the chimera with 3 glycines. The forward 5′-AGTGGAGCCCCTAGGTCAGGCGGTGGCGGTGGCAAAAAGGCAGAAAACGCTCATAAT-3′ and reverse 5′-GCTCATGTTTGACAGCTTATCATCGATAAGCTTTTACCC-3′ primers were used for the 5-glycine chimera. The forward primers were synthesized with an overhanging *Hind III* restriction site upstream of the BMR start codon, whereas the reverse primers contained an *Avr II* site downstream of the stop codon. The underlined letters represent the endonuclease restriction sites. DNA was melted for 2 min at 94°C, followed by 30 cycles of 1 min at 94°C, 1 min and 45 s at 60°C, 1 min at 72°C. The polymerase chain reaction products obtained were digested with *Hind III* and *Avr II* endonucleases and purified by the GeneClean II method as described by the manufacturer. The pCW-3A4-BMR vector was digested for 1 h at 37°C with *Hind III* and *Avr II* and genomic DNA corresponding to pCW-3A4 was isolated by NAGEL KIT extraction and then ligated using T4 DNA ligase with the sticky sites of the digest PCR products. The ligation mix was transformed into *E. coli* DH5α cells and plated on LB agar containing 100 μg/ml ampicillin. The positive clones were chosen and the plasmid DNA was purified using a Mini-prep plasmid extraction kit (Sigma, Italy). The correct insertion of the glycine codons was verified by DNA sequencing.

### Expression, purification, and characterization

Heterologous expression of 3A4-BMR, 3A4-3GLY-BMR, and 3A4-5GLY-BMR was performed by transforming competent *E. Coli* DH5α cells with the corresponding plasmid vectors. The proteins were purified using diethylaminoethyl and hydroxylapatite columns as described before (Dodhia et al., 2006). The purity of the enzymes was checked by sodium dodecyl sulfate polyacrylamide gel electrophoresis on 7.5% gel using a Mini-Protean apparatus (Bio-Rad, USA) with Coomassie blue staining.

Protein concentrations were determined from the absorption at 450 nm of the carbon monoxide-bound reduced form by using the Omura and Sato method (Omura and Sato, 1964) using an extinction coefficient of 91 mM^−1^ cm^−1^. Protein reduction was achieved by addition of saturating sodium dithionite, and formation of the carbon monoxy-adduct of the heme was obtained by bubbling the reduced enzyme with carbon monoxide.

### Determination of the heme and flavin contents

The ratio protein:heme:FMN:FAD were calculated using different spectroscopic methods. Protein concentration was estimated using Bradford assay (Bradford, 1976), while the heme concentration was obtained from the CO-binding assay described above (Omura and Sato, 1964). FAD and FMN contents were calculated using fluorescence emission spectroscopy in each purified enzymes (Faeder and Siegel, 1973). Excitation at 450 nm produced fluorescence emission spectra with an emission maximum at 535 nm as expected for flavin-containing proteins. Quantification of FAD and FMN present in the protein is based on the different behavior of the two cofactors at different pH-values (Aliverti et al., 1999). The samples were boiled for 3 min to release the flavins, then rapidly cooled on ice and centrifuged at 14,000 g for 10 min. An aliquot of the clear supernatant was diluted with standard buffer (0.1 M potassium phosphate buffer pH 7.7, containing 0.1 mM EDTA) so that the final volume was 1.5 mL and the mixture was transferred to a 1 cm path-length fluorimeter cuvette.

Fluorescence emission was recorded at 535 nm; the excitation wavelength was set at 450 nm. After the initial fluorescence measurement at pH 7.7, the pH of the solution was adjusted to 2.6 by the addition of 0.1 mL of 1 N HCl (final concentration 62.5 mM) and the fluorescence was determined again. The concentrations of FAD and FMN in the supernatant were determined from the fluorescence intensity compared to those obtained for standard solutions of flavins by using as extinction coefficients 11.3 and 12.2 mM^−1^cm^−1^, respectively (Aliverti et al., 1999).

### Time dependent loss of CO-binding

The stability of 3A4-BMR fusion proteins was compared with that of CYP 3A4 by following the loss of CO-binding at a temperature of 37°C. Recombinant CYP3A4 was expressed and purified as described by Dodhia et al. (2006). CO-binding spectral assay was performed at 37°C both in the presence of CYP3A4 and 3A4-BMR chimeric proteins as described in the Section Determination of the Heme and Flavin Contents by using 1.6 μM protein solutions. The absorbance at 450 nm was followed for 60 min by collecting one spectrum every 5 min at 37°C. All the collected spectra were corrected by subtraction of the spectrum of the reduced protein and the loss of CO-binding was measured as a percentage loss in absorbance of the peak at 450 nm.

### Anaerobic stopped flow spectrophotometric evaluation of NADPH consumption

Rates of NADPH oxidation were measured using a Hi-Tech scientific SF-61 single mixing stopped-flow system (TgK Scientific, UK) at 25°C in a glovebox (Belle Technology, UK) with an oxygen concentration below 10 ppm. NADPH consumption was monitored at 340 nm (ε = 6.22 mM^−1^ cm^−1^) for 750 sunder anaerobic conditions. Proteins and reducing agent solutions (in the absence and presence of saturating amounts of substrate) were prepared in syringes in an anaerobic glove box and transferred to the sample handling unit of the stopped-flow instrument. The experiment was performed by mixing the NADPH (syringe A, 200 μM NADPH in 50 mM potassium phosphate buffer pH 8.0) and the enzyme or enzyme plus substrate solutions (syringe B, 1 μM enzyme in 50 mM potassium phosphate buffer pH 8.0+150 μM erythromycin or testosterone). Analysis of the resulting kinetic data was performed with Kinetic studio V3 software (TgK Scientific, UK). In order to calculate the NADPH consumption rate, the plot of absorbance at 340 nm vs. time was fitted to a single exponential decay function.

### Determination of the reduction potentials of the chimeras

Potentiometric titrations were performed in an anaerobic glovebox (Belle Technology, UK) with an oxygen concentration below 10 ppm using a cuvette equipped with a gold laminar working electrode and an Ag/AgCl reference electrode connected to a potentiostat controlled by GPES3 software (Ecochemie, The Netherlands). Ambient reduction potentials were adjusted by addition of aliquots (2–5 μL) of anaerobically freshly prepared sodium dithionite solution. Titrations were performed in 50 mM potassium phosphate, pH 8. Electrode-solution mediation was facilitated by adding the following mediators at 5 μM concentration: methyl viologen (*E*_m_ = −440 mV), benzyl viologen (*E*_m_ = −359 mV), safranine O (*E*_m_ = −280 mV), anthraquinone-2,6-di sulfonic acid (*E*_m_ = −185 mV), phenazinemethosulfate (*E*_m_ = +80 mV), methylene blue (*E*_m_ = +11 mV). The modified cuvette was flushed with oxygen-free nitrogen before and during the measurements. After equilibration at each potential, the optical spectrum was recorded. Reduction of the heme in 3A4-BMR fusion proteins was followed by the increase in the absorption at 550 nm related to the merging of Q-bands, using a diode array spectrophotometer (TgK Scientific, UK). The fraction of reduced heme, proportional to the measured spectral transition, was plotted against potential, and the data were fitted to the Nernst equation:

$$\begin{array}{l} E=E_{m}+\left(RT/nF\right)ln\left(\left[ox\right]/\left[red\right]\right) \end{array}$$

where [*ox*] and [*red*] are the concentrations of oxidized and reduced heme, respectively, *E* is the solution reduction potential at equilibrium, *n* is the number of electrons, *E*_*m*_ is the midpoint potential, *F* is the Faraday constant, *R* is the gas constant, and *T* is the absolute temperature.

### Determination of the catalytic activity and coupling efficiency

The enzymatic activity of chimeras was determined toward erythromycin N-demethylation and testosterone 6β-hydroxylation.

Erythromycin N-demethylation was investigated under aerobic conditions by quantification of the formaldehyde arising from the N-demethylation of erythromycin using the Nash method (Nash, 1953), using a concentration range of this substrate under 120 μM where this molecule still acts as a substrate. Uncoupling reactions leading to hydrogen peroxide were determined and quantified using the N,N-dimethylaniline/4-amino-2,3-dimethyl-1-phenyl-3-pyrazolin-5-one/crude horseradish peroxidase colorimetric assay as described by Sugiura et al. (1977). Reactions were carried out at 37°C in a 1 cm path length quartz cuvette and consisted of the following mixture: 0.1 M potassium phosphate buffer pH 8.0, 2% glycerol, 5 mM MgCl_2_, 100 mM KCl, 1 μM of the chimeric protein and different concentrations of erythromycin in the range 0–120 μM. For each protein preparation, some preliminary experiments were performed in order to assess linearity of reaction rates with respect to time. After blanking the absorbance at 412 nm with the erythromycin incubated at 37°C for 5 min with 1 μM of the enzymes, catalysis was initiated by addition of 150 μM NADPH. The reactions were then stopped after 30 min by addition of HCl. The samples were centrifuged at 13,000 rpm for 5 min and 0.5 mL of the supernatant were transferred to another tube and mixed with 0.5 mL of a concentrated Nash reagent (3 g ammonium acetate, 40 μL acetylacetone, 60 μL acetic acid in 10 mL of water) as described before (Dodhia et al., 2006).

The reaction linearity was assessed over a 40 min time. Thus, the mixture was incubated at 50°C for 30 min and the samples were analyzed by measuring absorbance at 412 nm to determine the formation of formaldehyde. The amount of formaldehyde produced during the reaction was quantified by using a calibration curve constructed in the same reaction condition with a range of concentration between 0 and 30 μM. Zero-time controls were performed in the absence of erythromycin.

Coupling efficiency was calculated as the ratio of formaldehyde produced during erythromycin N-demethylation and the total amount of NADPH consumed by the chimeras.

Testosterone 6β-hydroxylation was investigated at 37°C by incubating 1 μM protein in the presence of testosterone in a concentration range between 1 and 150 μM for 5 min. Catalysis was initiated by addition of 200 μM NADPH. The reaction linearity was assessed over a 40 min time. The reactions were then stopped after 30 min by addition of HCl. The samples were centrifuged at 13,000 rpm for 5 min. The amount of 6β-hydroxytestosterone formed was evaluated by HPLC coupled with a diode array UV detector (Agilent-1200, Agilent technologies, USA) equipped with a 4.6 × 250 mm, 5 μm C18 column. Testosterone and 6β-hydroxytestosterone were separated using a gradient elution programmed as follows: isocratic elution of 20% acetonitrile and 80% water, 0–2 min; linear gradient elution from 20 to 90% acetonitrile and from 80 to 10% water, 2–20 min; linear gradient elution from 90 to 20% acetonitrile and from 10 to 80% water, 20–22 min; isocratic elution of 20% acetonitrile and 80% water 22 min-end of the run. The flow rate was set at 1 mL/min and detection wavelength was 244 nm. Retention times were 9.2 min and 13.1 min for 6β-testosterone and testosterone, respectively. Coupling efficiency was calculated as the ratio of 6β-hydroxytestosterone produced and the total amount of NADPH consumed by the chimeras.

### Measurement of cytochrome *c* reduction by the BMR of the chimeras

Cytochrome *c* reduction activity spectrophotometric assay was performed as described by Guengerich et al. (2009) with slight modification. In particular, 10 μM of cyt *c* (in 0.1 M potassium phosphate buffer pH 8.0) were pipetted into a 0.5 mL quartz cuvette (1 cm path-length). Then, 0.1 μM of chimeric proteins were added and gently mixed. The assay was started by adding NADPH to a final concentration of 75 μM. Using the kinetic mode of the spectrophotometer, the absorbance increase at 550 nm upon cytochrome *c* reduction was monitored for 5 min. The rate of cytochrome *c* reduction was calculated using reduced cyt *c* extinction coefficient at 550 nm,7.04 mM^−1^cm^−1^, as nmol of reduced cyt *c* per min. The experiment was carried out both in the absence and in the presence of 20% glycerol.

### Statistical analysis

All collected data were recorded at least in triplicate and expressed as mean ± standard deviation. The comparison of the results obtained was performed by one way ANOVA and two way ANOVA followed by Student-Newman-Keuls *post hoc* test using SigmaPlot software.

## Results

### Protein purification and UV-visible properties of the P450 3A4-BMR chimeras

**Figure 3:**
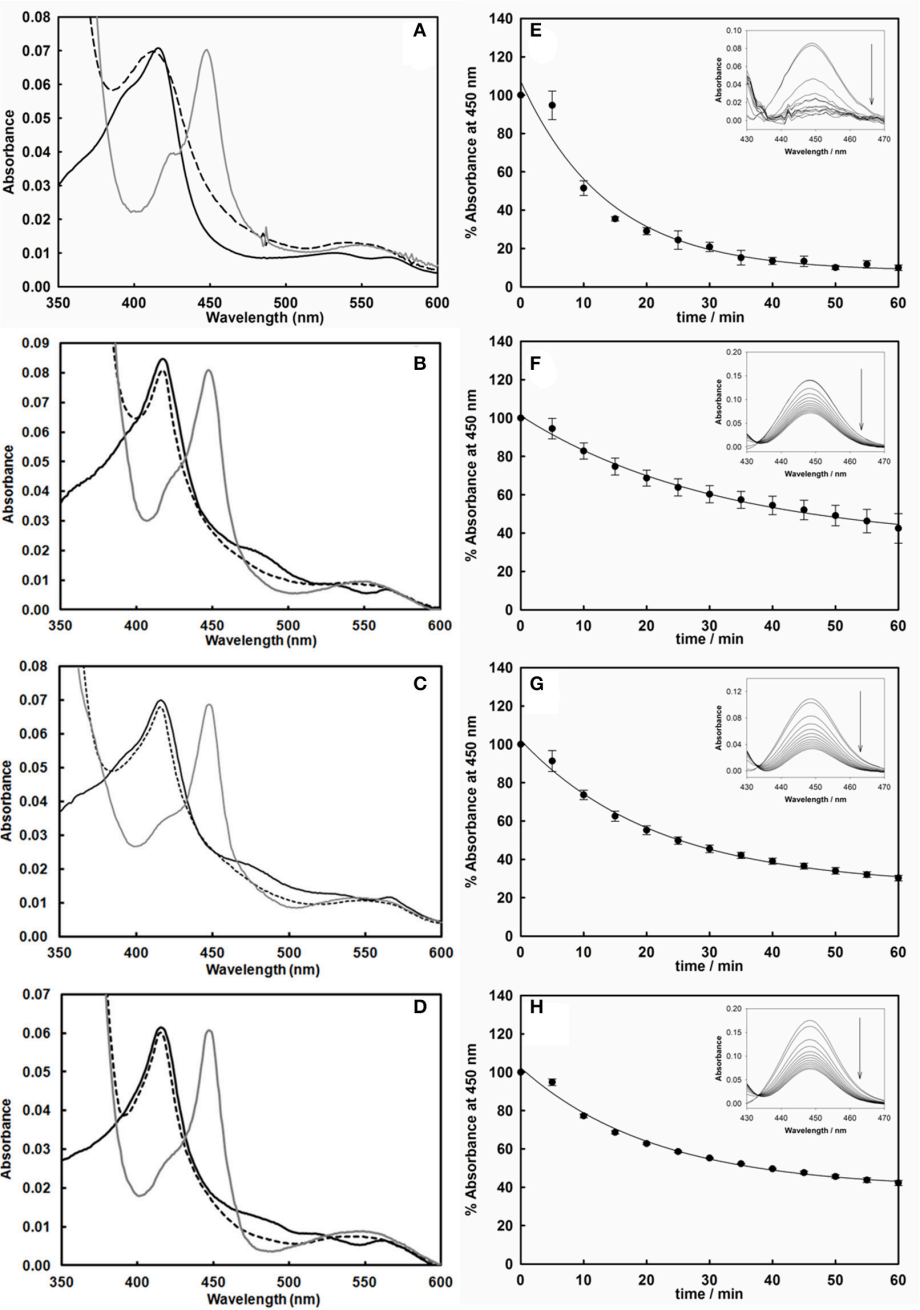
**Left: UV-vis spectra of the purified cytochrome P450 3A4 (A)**, 3A4-BMR **(B)**, 3A4-3GLY-BMR **(C)**, and 3A4-5GLY-BMR **(D)** in their oxidized (black line), reduced (dashed line), and reduced/carbon monoxide-bound (dark gray line) forms. Sodium dithionite completely reduces the flavin cofactors in all enzymes, leading to spectral bleaching. Binding of carbon monoxide results in the formation of Fe^2+^-CO complex with heme Soret absorption maxima shifted to 450 nm in all cases. Right: Time-dependent decrease of the absorbance at 450 nm of the CO-bound cytochrome P450 3A4 **(E)**, 3A4-BMR **(F)**, 3A4-3GLY-BMR **(G)**, and 3A4-5GLY-BMR **(H)**. CO binding loss is significantly higher, in terms of both rate and total absorbance decrease, for CYP 3A4 compared to the three BMR linked chimeras. Insets: difference spectra generated by subtraction of the reduced P450 spectrum to each Fe^2+^-CO complex spectrum.

After expression and purification (Figure 2B), the UV-visible spectral properties of the purified chimeras were analyzed between 300 and 600 nm for the oxidized, reduced and carbon monoxide bound forms. The spectra show the typical features of both heme and flavin cofactors (Figures 3A–D). These comprise the characteristic Soret peak at 418 nm corresponding to the oxidized heme iron, the β and α bands at 535 and 570 nm, the shoulder around 455–485 nm belonging to the oxidized FAD and FMN of the BMR domain.

Full reduction of the chimeras was achieved by addition of excess sodium dithionite leading to the hydroquinone forms with a concomitant decrease in the intensity in the visible region spectra (Figures 3A–D, dashed lines). In all cases a clear shift to 450 nm upon CO bubbling indicates the presence of a properly folded P450 domain (Figures 3A–D, dark gray lines).

The correct stoichiometry of the heme and flavin incorporation in the chimeras was also investigated by absorption and fluorescence spectroscopy. The heme content was determined from the absorbance value at 450 nm of the CO-adduct and the amount of the total protein was obtained by Bradford method. In all chimeras the ratio heme:protein resulted to be close to 1:1 (Table 1). This indicates that the proteins have a full complement of bound heme cofactor. The FAD and FMN content was determined by fluorescence emission spectroscopy as detailed in the materials and methods section, resulting in a FAD-to-protein and FMN-to-protein ratio of 0.81 and 0.82 for 3A4-BMR, 0.83, and 0.87 for 3A4-3GLY-BMR and 0.83 and 0.83 for P450 3A4-5GLY-BMR (Table 1). The ratios in flavins:heme content suggest that there is a minimal amount of apoenzyme with a nearly-stoichiometric amount of each cofactor and indicating that the domains are independently able to uptake the cofactor while folding.

**Table 1:** **Heme and flavin contents obtained for 3A4-BMR, 3A4-3GLY-BMR, and 3A4-5GLY-BMR**.

	**3A4-BMR**	**3A4-3GLY-BMR**	**3A4-5GLY-BMR**
Heme content (nmolheme/nmol protein)	0.97 ± 0.03	0.98 ± 0.01	0.98 ± 0.02
FMN content (nmol FMN/nmol protein)	0.82 ± 0.01	0.87 ± 0.03	0.83 ± 0.05
FAD content (nmol FAD/nmol protein)	0.81 ± 0.04	0.83 ± 0.02	0.83 ± 0.02

### NADPH consumption and reduction potentials of the heme

Since the inter-domain interactions modulated by the loops are crucial in determining both the electron transfer rates and reduction potentials, these parameters were evaluated on the three chimeras.

**Figure 4:**
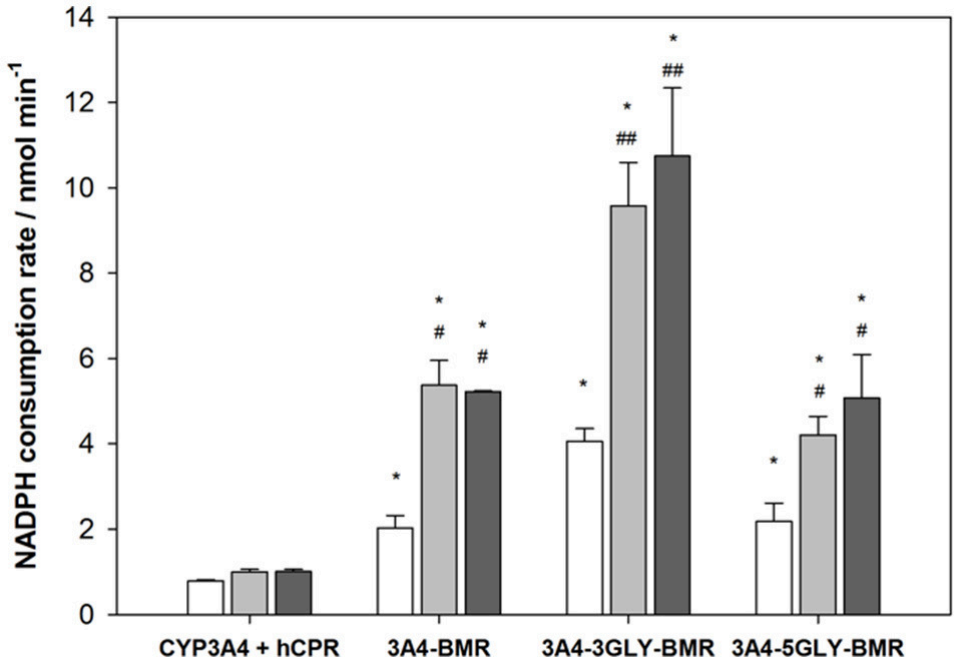
**Anaerobic NADPH oxidation rates for cytochrome P450 3A4-BMR, 3A4-3GLY-BMRand 3A4-5GLY-BMR compared to those obtained for CYP 3A4 + hCPR system, obtained both in absence (white bars) and in presence of substrate erythromycin (light gray) and testosterone (dark gray)**. Data are mean ± SD for 3 to 6 replicates. ^*^P < 0.001 compared to CYP 3A4 + hCPR system; ^#^*P* < 0.05, ^##^*P* < 0.001 compared to the protein in absence of substrate; two way ANOVA.

Rates of NADPH oxidation by the chimeras were measured by rapid scanning stopped flow spectrophotometry by following the decrease in absorbance at 340 nm for 750 s in anaerobic condition (Sevrioukova et al., 1996; Davydov et al., 2010), as this is the first essential step in the electron transfer chain involving the FAD and FMN cofactors of BMR to sustain the 3A4 catalysis (Guengerich, 1983). The NADPH consumption rates were estimated by fitting the absorbance decrease with single exponential decay equation both for 3A4-BMR chimeras and for CYP3A4 in the presence of hCPR as reference system. Moreover, NADPH oxidation rates were calculated also in the presence of substrate, erythromycin and testosterone, in saturating conditions. The results are summarized in the Figure 4. As reported, compared to CYP3A4/hCPR system, 3A4-BMR chimeric proteins were found significantly faster both in absence and in presence of substrate, resulting more efficient in the transfer of reducing equivalents from NADPH. In addition, 3A4-3GLY-BMR protein resulted significantly faster in NADPH consumption with respect to the other two constructs (*P* < 0.05), both in the presence and in the absence of substrate. Moreover, the presence of both erythromycin and testosterone significantly enhances NADPH consumption rate in all the examined fusion proteins. This effect is probably due to the fact that the substrate-bound complex has a higher reduction potential and is capable to accept electrons from the redox partner more easily leading to a faster reduction of the heme-Fe^3+^ to heme-Fe^2+^ (Sligar et al., 1979; Guengerich, 1983).

Reduction potentials of the heme were determined with anaerobic spectro-potentiometric titrations of the chimeras using the method described by Dutton (1978). The change in population from the oxidized (Fe^3+^) to the reduced (Fe^2+^) state upon addition of sodium dithionite reducing agent was monitored by observing the spectral transition related to the merging of the Q-bands in a single band at 550 nm. Reduction potential values were calculated by plotting the calculated 3A4-BMR reduced fraction vs. the recorded potential using the Nernst's equation. Midpoint potential values were calculated to be −367±3 mV, −327±8 mV, and −397±8 mV for 3A4-BMR, 3A4-3GLY-BMR and 3A4-5GLY-BMR, respectively.

The differences observed in NADPH oxidation and reduction potentials indicate that the loops indeed have an impact on these parameters and therefore also different functional performace in catalysis are expected.

### Stability of the chimeras

In order to study if and how the length of the loop has an impact on the stability of the P450 catalytic module, the time dependent decrease of the absorbance at 450 nm upon reduction and CO binding was used to compare the relative stability of P450 proteins (Lin et al., 2005; Foti et al., 2011; Amunugama et al., 2012). This assay is based on the assumption that the loss of the band at 450 nm is associated with a loss of activity of P450 enzymes (Omura and Sato, 1964). Thus, the absorbance at 450 nm was followed for 60 min at 37°C after the CO-binding assay for CYP 3A4 as well as the three 3A4-BMR fusion proteins. The results are shown as percentage residual absorbance in the Figures 3E–H. Figure 3E shows that 3A4 domain when alone retains only 10 ± 1% of the initial absorbance at 450 nm with a loss of 450 nm peak rate of 0.072 ± 0.011 min^−1^, whereas 3A4-BMR (Figure 2F), 3A4-3GLY-BMR (Figure 2G), and 3A4-5GLY-BMR (Figure 2H) show a higher stability with a residual absorbance at 450 nm of 42 ± 7, 30 ± 1, and 42 ± 1% respectively and a loss of 450 nm peak rate of 0.032 ± 0.003 min^−1^, 0.046 ± 0.004 min^−1^, and 0.047 ± 0.005 min^−1^ respectively. These values show that, in these experimental conditions, the stability is significantly higher for the 3A4-BMR chimeras (*P* < 0.001, one way ANOVA) when compared to the 3A4 alone.

### Catalytic properties and coupling efficiency of the chimeras

**Figure 5:**
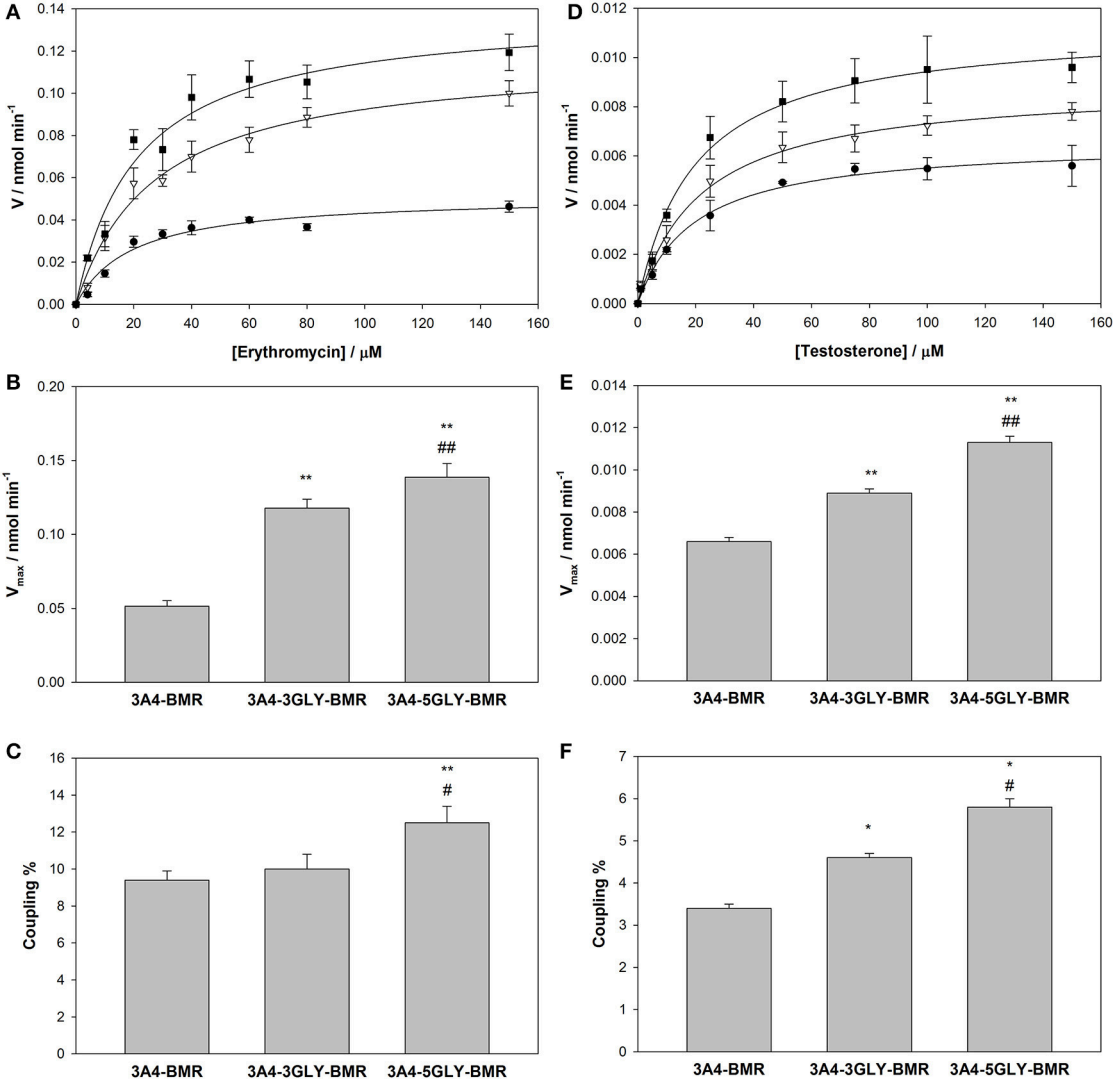
**Activity and coupling of the 3A4-BMR, 3A4-3GLY-BMR and 3A4-5GLY-BMR chimeras toward erythromycin N-demethylation. (A)** Catalytic activity measured as HCHO production rate in the presence of 3A4-BMR (circles), 3A4-3GLY-BMR (triangles) and 3A4-5GLY-BMR (squares) as function of erythromycin concentration fitted with MichaelisMenten curves. **(B)** Summary of V_max_-values calculated during erythromycin N-demethylation in the presence of cytochrome P450 3A4-BMR, 3A4-3GLY-BMR and 3A4-5GLY-BMR. **(C)** Summary of coupling percentage values calculated during erythromycin N-demethylation in the presence of cytochrome P450 3A4-BMR, 3A4-3GLY-BMR and 3A4-5GLY-BMR. **(D)** Catalytic activity measured as 6β-hydroxytestosterone production rate in the presence of 3A4-BMR (circles), 3A4-3GLY-BMR (triangles) and 3A4-5GLY-BMR (squares) as function of testosterone concentration fitted with Michaelis Menten curves. **(E)** Summary of V_max_-values calculated during testosterone 6β-hydroxylation in the presence of cytochrome P450 3A4-BMR, 3A4-3GLY-BMR and 3A4-5GLY-BMR. **(F)** Summary of coupling percentage values calculated during testosterone6β-hydroxylation in the presence of cytochrome P450 3A4-BMR, 3A4-3GLY-BMR and 3A4-5GLY-BMR. All reactions were carried out at 37°C for 30 min. Data are mean ± SD of at least 3 replicates. *R*^2^> 0.99. ^*^P < 0.01, ^**^P < 0.001 compared to 3A4-BMR, ^#^*P* < 0.05, ^##^*P* < 0.001 compared to 3A4-3GLY-BMR, one way ANOVA.

Turnover of the different chimeras was studied in the presence of the substrates erythromycin and testosterone using NADPH as electron source, and the results are reported in Table 2. Quantification of both the amount of product formed in the N-demethylation of erythromycin and coupling efficiency, determined from the ratio product formed/NADPH oxidized, were performed for the three chimeras. Erythromycin N-demethylation rates were calculated at increasing concentration of the substrate in the presence of all the 3A4-BMR constructs, and the experimental data were fitted to the Michaelis-Menten model in order to calculate kinetic parameters (Figure 5A). The K_M_-values of 3A4-BMR, 3A4-3GLY-BMR, and 3A4-5GLY-BMR were 20.8 ± 3.6, 27.4 ± 4.0, and 21.3 ± 4.4 μM respectively. In this case no statistically relevant differences were found. These results show that the glycine linker length does not influence 3A4-BMR affinity for erythromycin substrate, indicating that the binding pockets of the heme domain of the chimeras are not perturbed.

**Table 2:** **Catalysis and uncoupling parameters for erythromycin N-demethylation and testosterone 6β-hydroxylation for 3A4-BMR, 3A4-3GLY-BMR, and 3A4-5GLY-BMR**.

	**Erythromycin N-demethylation**			**Testosterone 6β-hydroxylation**		
	**K_M_(μM)**	**V_max_(nmol min^−1^)**	**Coupling efficiency%^(a)^**	**K_M_(μM)**	**V_max_ (nmol min^−1^)**	**Coupling efficiency%^(b)^**
3A4-BMR	20.8 ± 3.6	0.0515 ± 0.0004	9.4 ± 0.5	19.6 ± 2.6	0.0066 ± 0.0002	3.4 ± 0.1
3A4-3GLY-BMR	27.4 ± 4.0	0.1177 ± 0.0006	10.0 ± 0.8	21.3 ± 2.7	0.0089 ± 0.0002	4.6 ± 0.1
3A4-5GLY-BMR	21.3 ± 4.4	0.1387 ± 0.0009	12.5 ± 0.9	19.8 ± 2.2	0.0113 ± 0.0003	5.8 ± 0.2

*Coupling parameters are determined at saturating erythromycin (120 μM) and testosterone(150 μM) concentrations. Values are means ± standard deviation of four experiments*.

(a) *The coupling efficiency is calculated as: HCHO × 100/NADPH consumed*.

(b) *The coupling efficiency is calculated as 6β-hydroxytestosterone × 100/NADPH consumed*.

Further analysis in terms of turnover rate led to V_max_-values of 0.0515 ±.0004, 0.1177 ± 0.0006, and 0.1387 ± 0.0009 nmol min^−1^ for 3A4-BMR, 3A4-3GLY-BMR, and 3A4-5GLY-BMR (Figure 4B), respectively. In this case, a statistically significant increase in V_max_ for 3A4-3GLY-BMR and 3A4-5GLY-BMR with respect to the 3A4-BMR construct was observed. This shows that the glycine linker increases the enzyme turnover rate in the presence of erythromycin substrate. Moreover, a significant increase in V_max_-value was found for 3A4-5GLY-BMR compared to 3A4-3GLY-BMR.

Also the testosterone 6β-hydroxylation rates were calculated at increasing concentrations of the substrate in the presence of all the 3A4-BMR constructs, and the results were fitted to the Michaelis-Menten model in order to calculate kinetic parameters (Figure 5D). The K_M_-values obtained from fitting the Michaelis-Menten curves of 3A4-BMR, 3A4-3GLY-BMR, and 3A4-5GLY-BMR were 19.6 ± 2.6, 21.3 ± 2.7, and 19.8 ± 2.2 μM respectively, indicating again no statistically relevant differences in the binding pocket. The V_max_-values were 0.0066 ± 0.0002, 0.0089 ± 0.0002, and 0.0113 ± 0.0003 nmol min^−1^ respectively for 3A4-BMR, 3A4-3GLY-BMR, and 3A4-5GLY-BMR enzymes (Figure 5E). As observed in the presence of erythromycin, when comparing V_max_ results, a statistically significant increase was found for 3A4-3GLY-BMR and 3A4-5GLY-BMR with respect to the 3A4-BMR construct. Also in this case the glycine linker increases the enzyme turnover rates, with a significant higher V_max_-value for 3A4-5GLY-BMR compared to 3A4-3GLY-BMR.

The coupling efficiency percentage during erythromycin N-demethylation, calculated as ratio of HCHO formed and NADPH consumed, were found to be 9.4 ± 0.5, 10.0 ± 0.8, and 12.5 ± 0.9% for 3A4-BMR, 3A4-3GLY-BMR, and 3A4-5GLY-BMR, respectively (Figure 5C). When performing statistical analysis of these results, the 3A4-5GLY-BMR fusion protein was found more coupled than the other two constructs, whereas no significant difference was found when comparing 3A4-BMR and 3A4-3GLY-BMR. A similar comparison was also performed for testosterone catalysis by calculating coupling efficiency as the ratio of the amount of 6β-hydroxytestosterone formed and NADPH consumed. The calculated coupling efficiency percentage ratios were found to be 3.4 ± 0.1, 4.6 ± 0.1, and 5.8 ± 0.2% for 3A4-BMR, 3A4-3GLY-BMR, and 3A4-5GLY-BMR, respectively (Figure 5F).

### NADPH–cytochrome *c* reduction by 3A4-BMR chimeras

The results from enzymatic activity, coupling and reduction potentials indicate that the different loops affect the interaction between the BMR and 3A4 domains of the chimeras, probably due to the different degrees of length and flexibility associated with them. Cytochrome *c* was therefore used as external probe to check its ability to intercept the electrons of NADPH delivered by the BMR when this is exposed and hence available to interact with proteins other than the 3A4 domain. The ability of cytochrome *c* to be reduced by NADPH *via* free cytochrome P450 reductase has already been described in the literature (Guengerich et al., 2009).

The capability of 3A4-BMR chimeras to use NADPH electrons to reduce cytochrome *c* is strictly related to cytochrome *c* ability to interact directly with the BMR domain when this is exposed. In order to explore the effect of viscosity on the interaction of cytochrome *c* with the chimera BMR domain, we performed the experiments both in the absence and in the presence of 20% glycerol. The reduction rates were calculated as nmoles of reduced cytochrome *c* per min and the results are summarized in the Figure 5.

The 3A4-5GLY-BMR chimera shows the highest cytochrome *c* reduction rate compared to the other two proteins. Moreover, the presence of 20% glycerol significantly impaired cytochrome *c* reduction ability of all the three constructs, with the 3A4-5GLY-BMR being the most affected one.

## Discussion

Cytochromes P450 have been extensively used in our laboratories to investigate their potential for applications in biocatalysis as electron transfer domains in chimeric constructs (Cunha et al., 1999; Sadeghi et al., 2011; Sadeghi and Gilardi, 2013). This work investigates how the stability and activity of self-sufficient chimeric human enzymes can be optimized not only by creating fusion proteins but also with the correct choice of loop connecting the reductase and the catalytic domains.

The stability of the P450 catalytic domain is significantly improved in the presence of the BMR domain since all the three fusion proteins showed a 1.5–2 fold lower rate of P450 loss compared to P450 3A4. In this case, the presence of an extra domain rather than the length of the loop is essential for stability as no significant differences are observed among the three proteins. However, the length of the loop has an impact on other parameters, such as NADPH oxidation rates and reduction potential. In particular, the chimeric proteins show a higer NADPH consumption rate compared to the CYP3A4-hCPR system at a 1:1 molar ratio. Moreover, the 3A4-3GLY-BMR protein shows a 2-folds faster rate in NADPH consumption with respect to the other two constructs. This effect suggests that the 3GLY construct is more prone to accept electrons from the redox partner with respect to no GLY and 5GLY leading to a faster reduction of the heme-Fe^3+^ to heme-Fe^2+^ (Sligar et al., 1979). These data show that there is a critical length of the linker between the domains that can modulate the inter-domain interactions that determine the rate of the electron transfer.

The fastest oxidation rate in the 3GLY protein is explained by its more positive reduction potential compared to the other two fusion proteins. It is known that in redox proteins, the interaction with the redox partner can influence the conformation, the reduction potential and in general, the dielectric environment of the redox cofactor (Bendall, 1996). Here, the three fusion proteins show a different, although negative, reduction potential that can reflect a different dielectric heme environment triggered by a different domain arrangement and interaction.

When investigating the catalytic domain ability to use reducing equivalents for erythromycin N-demethylation and testosterone 6β-hydroxylation during the catalytic event, the 5GLY construct resulted the best for both turnover rate and coupling efficiency. Thus, the increase in turnover number and coupling efficiency is neither directly related to an improvement in electron transfer rate nor to a more positive heme iron reduction potential. Differences in substrate binding can also be excluded as the K_M_-values measured for both erythromycin and testosterone substrates are similar for the three proteins and within the range of previously published data (Riley and Howbrook, 1998; Yuan et al., 2002). Moreover, these data are consistent with previously published results in which it was demonstrated that the use of artificial redox chains allows the regulation of the electron flow to the P450 catalytic domain through the mediation of the non-physiological reductase domain by lowering the electron transfer rate and improving the correct electrochemical inter-domain tuning thus enhancing coupling efficiency and catalytic activity (Dodhia et al., 2008; Degregorio et al., 2011; Castrignanò et al., 2014).

**Figure 6:**
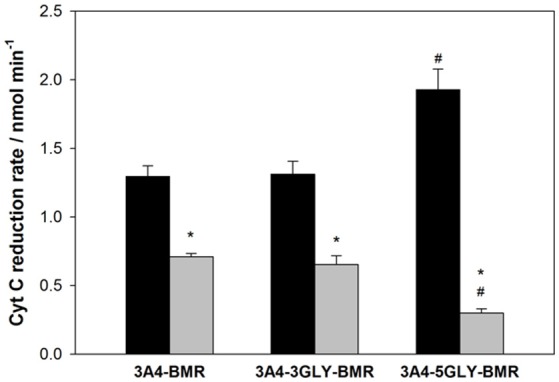
**Summary of NADPH-cytochrome ***c*** reduction rates by 3A4-BMR, 3A4-3GLY-BMR and 3A4-5GLY-BMR chimeras**. Data obtained in the presence of 0% glycerol (black bars) and 20% glycerol (gray bars) are shown. They are mean ± SD of at least 3 replicates. ^*^P < 0.001 vs. 0% glycerol; ^#^*P* < 0.001 vs. 3A4 BMR and 3GLY(two way ANOVA followed by Student-Newman-Keuls *post hoc* test).

In order to investigate if there is a correlation between the observed improved turnover/coupling efficiency and an increased inter-domain flexibility in the 5GLY protein, cytochrome *c* was used as external probe to check its ability to intercept the electrons of NADPH delivered by the BMR when this is exposed and hence available to interact with proteins other than the 3A4 domain. The 5GLY resulted in the fastest in cytochrome *c* reduction and this result can be explained by the highest flexibility between the reductase and catalytic domain of the chimera allowing a better interaction with cytochrome *c* and an easier electron delivery from BMR reductase domain (Figure 6). This interpretation is further confirmed by the fact that the impairment of inter-domain flexibility upon glycerol addition that increases the medium viscosity, causes a significant decrease in the cytochrome *c* reduction rate in all the three constructs, with a more marked effect on the more flexible for 5GLY loop.

**Figure 7:**
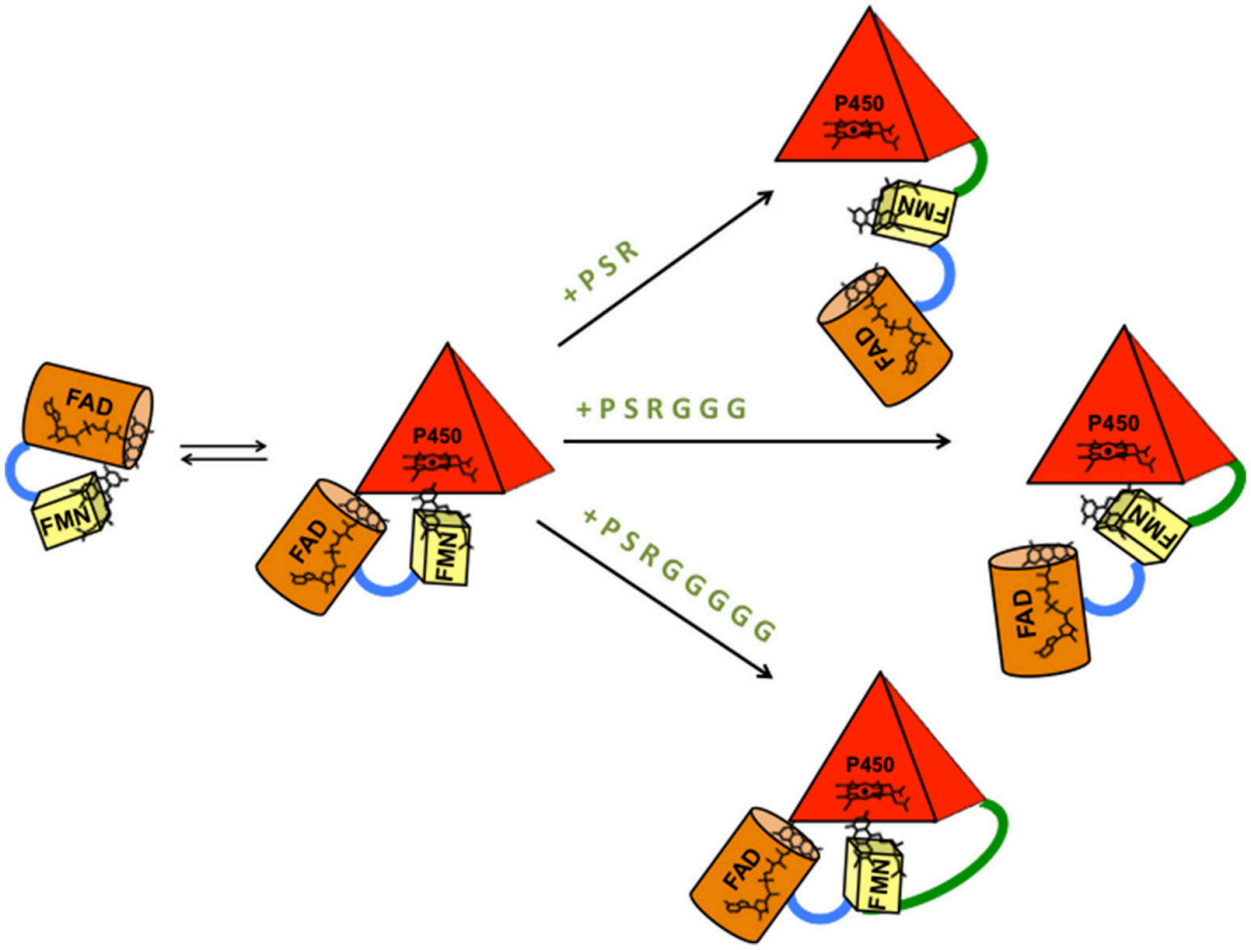
**Model of BMR-P450 active complex formation and interaction in the three different chimeric proteins**. The length of the inter-domains loop favors the formation of different types of complexes that can justify different electron transfer rates, turnover and coupling efficiency. P (proline), S (serine), and R (arginine) are the three amino acids common to the three chimeras. G is glycine.

Taken together, the erythromycin N-demethylation and testosterone 6β-hydroxylation activity and the coupling results show that the 3A4-5GLY-BMR chimera is the more catalytically efficient and the more coupled. This result suggests that the higher linker flexibility allows a more proficient arrangement of catalytic and reductase domains and improves the tuning of the electron transfer to the active site leading to a more accurate electrons delivery from the NADPH electron source during the catalytic event (Figure 7).

## Conclusions

The creation of chimeric proteins was shown before to improve the solubility of P450 3A4 and to generate catalytically self-sufficient enzymes (Degregorio et al., 2011). Here, the P450 3A4-BMR catalytic performance is improved in terms of coupling efficiency and enzyme turnover by engineering the loop connecting the P450 3A4 and the BMR modules. The glycine linker acts as a flexible hinge that can control electron flow rate, catalytic activity and coupling efficiency of the 3A4-BMR chimeras. Data in this work highlight that the modulation of the length of the linker connecting the modules of a multi-domain protein has an impact on electron transfer, catalytic ability and coupling efficiency of the system that can be ascribed to the modulation of inter-domain flexibility and interactivity rather than to a different tuning of the electron transfer process. These parameters can be considered as the major requirement for an optimization of the electron delivery to the heme iron center showing that the design of the linker hinge between the interacting domains plays a crucial role in the optimization of reductase domain improvement of P450 catalytic efficiency. Our results suggest that for the 3A4-BMR fusion proteins studied here, the electron transfer may be achieved through the direct flow between the reductase and the heme domain rather than through “inter subunits” interaction between protomers in a dimeric system as suggested for the P450 BM3 system.

This molecular design approach can be successfully developed for the obtainment of performing enzymatic paradigms suitable for biotechnological applications. The next step is the introduction of such catalysts in bacterial cells for the large-scale production of drug metabolites using whole cells as a less expensive system.

## Author contributions

DD performed the catalysis experiments on Erythromycin. SD performed the catalysis experiments on Testosterone. SC performed the uncoupling experiments, redox potential determination and cyt c interaction. GDN and GC performed the modeling of the chimeras. SS and GG interpreted the data and wrote the manuscript.

### Conflict of interest statement

The authors declare that the research was conducted in the absence of any commercial or financial relationships that could be construed as a potential conflict of interest.
